# Frequency of Antimicrobial Resistance and Class 1 and 2 Integrons in *Escherichia Coli* Strains Isolated from Urinary Tract Infections

**DOI:** 10.22037/ijpr.2020.1101148

**Published:** 2020

**Authors:** Mahboobe Mirnezami, Reza Ranjbar, Mohammad Niakan, Mohammad Hossein Ahmadi

**Affiliations:** a *Department of Microbiology, Faculty of Medicine, Shahed University, Tehran, Iran. *; b *Molecular Biology Research Center, Systems Biology and Poisonings Institure Baqiyatallah University of Medical Sciences, Tehran, Iran.*

**Keywords:** Antimicrobial resistance, Class 1 and 2 integrons, *Escherichia coli* strains, Urinary tract infections, Antibiotic susceptibility

## Abstract

Resistance to antimicrobial compounds in *E. coli* strains is increasing. Integrons are mobile genetic elements that lead to the spread and transfer of antibiotic resistance genes in bacteria. The aim of the present study was to determine the frequency of class 1 and 2 integrons as well as the antimicrobial resistance in *E.coli* strains isolated from urinary tract infections (UTIs). A total of 100 clinical isolates of uropathogenic *E. coli* (UPEC) were collected from patients having UTIs. These strains were identified using biochemical tests. The antibiotic susceptibility patterns of the isolated bacteria were determined in accordance with the standard method recommended by the clinical and laboratory standards institute (CLSI). The presence of class 1 and 2 integrons was determined by PCR method. The most frequent antibiotic resistance was observed to ampicillin (72%), co-trimoxazole (66%), and nalidixic acid (62%). The highest sensitivity was seen to amikacine (11%) and gentamicin (20%). The multi-drug resistance (MDR) was observed in 80% of *E. coli* isolates. 70% and 3% of *E. coli* isolate possessed class 1 and 2 integrons, respectively. Our data suggest that the antimicrobial resistance to some antibiotics as well as the frequency of class 1 and 2 integrons is very high in *E. coli* strains. Moreover, class 1 integrons are correlated with resistance to ampicillin, gentamicin, ciprofloxacin, co-trimoxazole, and nalidixic acid. Therefore, it is very important to monitor integron-induced drug resistance, especially class 1 integron, in order to control the urinary tract infections causing by MDR *E.coli* strains.

## Introduction

Urinary tract infections (UTIs) are among the most frequent infectious diseases worldwide. UTIs comprise a range of disorders, including cystitis (infection of the bladder) and pyelonephritis (infection of the kidney) (1). *Escherichia coli* is the most predominant pathogen causing 80-90% of community-acquired UTIs and 30-50% of nosocomially-acquired UTIs (2). The development of antibiotic resistance has led to discovery of many natural mobile elements, including transposons and conjugative plasmids. Comparative sequence analysis of these elements has ultimately led to discovery of integrons (3) as elements which contain the genetic determinants of the components of a site-specific recombination system that recognizes and captures mobile gene cassettes (4). They are defined by the presence of an integrase gene (intI), a recombination site (attI), and one or two promoters responsible for expression of the inserted gene cassettes (5). Three classes of resistant integrons (RIs) have been defined on the basis of the divergence among their integrase genes, and each class appears to be able to acquire the same gene cassettes (3). Class 1 integrons are found extensively in clinical isolates, and most of the known antibiotic-resistance gene cassettes belong to this class. Class 2 integrons are exclusively associated with Tn7 derivatives and class 3 integrons are thought to be located in a transposon (6). 

Integrons are gene exchange systems and are known to play a significant role in the acquisition and dissemination of antimicrobial resistance genes and to be selected by antimicrobial pressure. These systems play a broad and important role in MDR *E. coli* strains (strains showing antimicrobial resistance to multiple antimicrobial drugs) (5). There is a need for improved surveillance for drug resistance and its mechanisms of dissemination and persistence and mobility of resistance genes in the community and clinical settings.

Considering the role of *E. coli* in urinary tract infections and increasing antibiotic resistance in this bacterium on the one hand and the importance of integrons in acquiring antibiotic-resistant gene cassettes on the other hand, the aim of this study was to investigate the antibiotic resistance and the frequency of class 1 and 2 integrons in *E. coli* strains isolated from urinary tract infections.

## Experimental

*Sample collection and antibiogram*

In this cross-sectional study, that was conducted over a period of six months from January 2016 to June 2016, a total of 100 *E. coli* isolates were collected from UTI samples of outpatients referring to Baqiyatallah Hospital, Tehran, Iran. UTI in patients was confirmed based on clinical signs and laboratory findings related to this infection. All strains were identified using conventional biochemical tests. The bacterial suspension was stored in TBS medium containing 20% ​​glycerol at -70 °C. The Antibiotic susceptibility pattern of strains was determined using disk diffusion method (Kirby-Bauer) on Mueller Hinton agar medium (Merck, Germany) according to the CLSI guidelines (7). The antibiotics disks used in this study were as follows: Ampicillin, amikacin, gentamicin, ceftazidime, cefotaxime, ciprofloxacin, co-trimoxazole, and nalidixic acid.

*DNA extraction and PCR*

**Figure 1:**
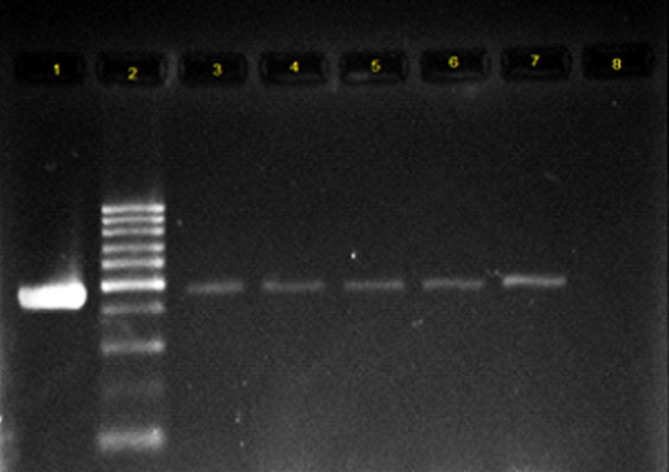
Electrophoresis of PCR product on 1.5% agarose gel for Int1 gene**.** Lane 1: positive control for Int1 gene; Lane 2: 100 bp DNA ladder as the molecular size marker; Lane 3-7: the Int1 gene detected in UPEC strains; Lane 8: PCR mix with no template (negative control).

**Figure 2:**
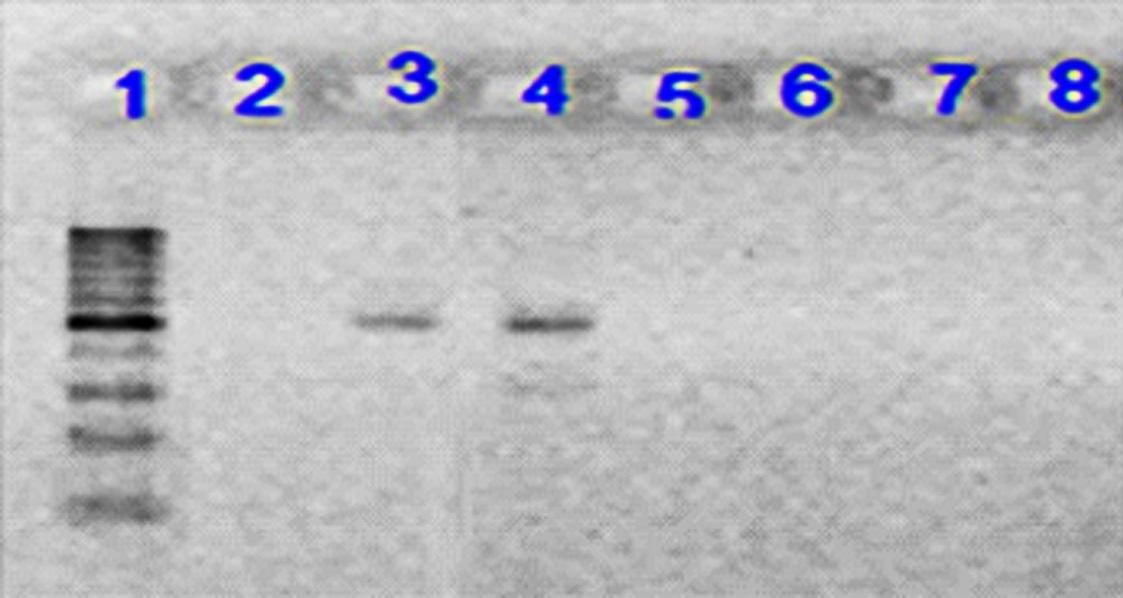
Electrophoresis of PCR product on 1.5% agarose gel for Int2 gene. Lane 1: 100 bp DNA ladder as the molecular size marker; Lane 2: PCR mix with no template (negative control); Lane 3: positive control for Int2 gene; Lane 4: the Int2 gene detected in UPEC strain; Lane 5-8: UPEC strains negative for Int2 gene

All the collected isolates were extracted using DNA extraction kit, a product of Bioneer Co. of Korea, according to the manufacturer’s instructions. PCR test was conducted to identify the genes coding for integrase enzyme. The reaction was done in a total volume of 20 μL, so that 1 μL of each primer, 1 μL of DNA template, 10 μL of Master Mix and 7 μL of distilled water were used. Primers used are shown in Table 1 (8, 9). Class 1 integron amplification was done in the following conditions: denaturation temperature of 95 °C for 5 min, 30 thermal cycles with denaturation temperature of 94 °C for 50 seconds, Primer binding temperature of 54 °C for 50 seconds, amplification temperature of 72 °C for 1 minute, and final amplification temperature of 72 °C for 5 min. Class 2 integron amplification conditions were similar to those of class 1 integron gene, but the annealing temperature was 52 °C. Electrophoresis of PCR products was performed on 1.5% agarose gel for 1 h in voltage of 80 volts. Then, they were stained using ethidium bromide. The results were observed using Gel Documentation system and examined under ultraviolet light. To validate the test, the confirmed control strains of *E. coli,* containing the integron gene were used as the positive control and the microtubes, containing the reaction material with no DNA template were used as PCR test contamination control (Figure 1 and 2). The PCR product sequencing was done by Bioneer Co. of Korea as ordered by Takapouzist Co. in Tehran.

*Statistical analysis*

 The data were analyzed using SPSS statistical software, version 22 (SPSS Inc., Chicago, IL). The association between presence of class 1 integron and antibiotic resistance was determined by Mann-whittney test. A *p*-value of > 0.05 was considered statistically significant.

## Results

The most frequent antibiotic resistance was observed to ampicillin (72%), co-trimoxazole (66%), and nalidixic acid (62%). The highest sensivity was seen to amikacin (11%) and gentamicin (20%). All strains were resistant to at least one antibiotic and MDR strains that were observed in 80% of *E.coli* isolates. Molecular analysis for the presence of class 1 and 2 integrons showed that 70% of the isolates had class 1 integron, while class 2 integron was observed only in 3% of the strains. The results of Mann-whittney test showed the statistically significant relationship between the presence of class 1 integron and resistance to ampicillin, gentamicin, ciprofloxacin, co-trimoxazole, and nalidixic acid. There was no significant association between the presence of class 1 integron and the resistance to amikacin, ceftazidime, and cefotaxime (Table 2).

## Discussion

The level of antibiotic resistance among hospital and community-acquired isolates has steadily increased and become a major global health problem. Antibiotic resistance pattern of isolates from UTI, which is one of the most frequent infectious diseases and most common infection in hospital and care institution, is also changing (10). In the present study, which was conducted on *E. coli *strains causing urinary tract infections, the most susceptibility was observed to ampicillin, co-trimoxazole, and nalidixic acid. The antibiotic resistance to ampicillin in Jahrom, Hamedan and in similar studies in Turkey, India, Taiwan, and Mexico has been reported as 80.2%, 61.9%, 73.3%, 90%, 82.9%, and 83.7%, respectively (11-16). After the ampicillin, co-trimoxazole was the second ranked antibiotic in terms of showing the antibiotic resistance as follows: Jahrom: 76%, Hamedan: 56.4%, and Turkey: 63.3%, which was similar to the present study (11, 13, 16). In the present study, the most effective antibiotics against *E.coli* isolates were amikacin and gentamicin so that only 11% and 20% isolates were resistant to amikacin and gentamicin, respectively. The resistance rate to amikacin and gentamicin was as follows: in Hamadan, 6.1% and 18.8%; in Turkey, 4.9% and 13.9%; and in Mexico, 1.7% and 23.9% (13, 14, 16).

In the present study, MDR strains were observed in 80% of *E.coli* isolates. The level of antibiotic resistance among *E. coli* strains causing urinary tract infection varies from country to country. For example, in the United States (2000), India (2012), and Korea (2015), the frequency of MDR strains was reported as 7.1%, 75%, and 21.9%, respectively (17-19). In recent years, a high percentage of MDR *E. coli* strains have been reported in different parts of Iran (11, 20-23). The aim of the present study was to investigate the role of integrons in antibiotic resistance, which plays a broad and important role in MDR *E. coli* strains. In the present study, class 1 and 2 integrons of *E.coli* isolates were detected in 70% and 3% of isolates, respectively. Previous studies in the other parts of the world also investigated the frequency of different classes of integrons. In a study conducted in 2008 by Farshad *et al*., 6.25% and 10.41% of strains had class 1 and class 2 integrons, respectively (24). In 2011, Rezaee *et al*. investigated the prevalence rate of class 1 and 2 integrons among 140 clinical isolates of *E. coli*. Their results showed that 22.5% and 5.08% of the isolates had class 1 and class 2 integrons, respectively (25). In a study conducted in 2013 by Ranjbaran *et al*., class 1 and class 2 integrons were detected in 86% and 8% of the isolates, respectively (26). In a study by Khorramrooz *et al*., the frequency of class 1 and 2 integrons was 52% and 2.5%, respectively (22). In previous studies in California, Pakistan and Syria, the frequency of class 1 integron were reported as 49%, 43.56%, and 54.6%, respectively (27-29). In a study in 2015, Lin *et al*. showed that 25 isolates of 162 isolates had class 1 integron. No class 2 integron was found in Malaysia (30). 

One of the limitations in the present study was that we were unable to determine gene cassettes carried in the intergrons coding the resistance to the mentioned antibiotics. However, our data indicate that there is a significant correlation between class 1 integrons and resistance to ampicillin, gentamicin, ciprofloxacin, co-trimoxazole, as well as nalidixic acid. The resistance to these antibiotics could be due to the presence of resistance gene cassettes in this class of integrons. In the cases where there was no significant correlation between the presence of class 1 integrons and the antibiotic resistance, the resulting resistance can be achieved through other ways such as the presence of the resistance genes on resistance plasmids and etc. It is recommended to conduct more detailed studies on the nature of integrons, the gene cassettes carried in the intergrons, and the other resistance mechanisms in these species to provide better therapeutics and control strategies in the future.

**Table 1:** Primers used for PCR

**Reference**	**Amplicon Size (bp)**	**Primer Sequence**	**Gene**
(8)	484	3'-GGGTCAAGGATCTGGATTTC –F: 5' 3'-ACATGGGTGTAAATCATCGTC- R: 5'	Int1
(9)	466	GCAAATGAAGTGCAACGC-3'–F: 5' 3'- ACACGCTTGCTAACGATG - R: 5'	Int2

**Table 2:** The correlation between presence of class 1 integeons and antibiotic resistance

Antibiotics	**Isolated having integrons (n = 70)**			**Isolates lacking integrons (n = 30)**			*p*-value*
	Susceptible NO (%)	Intermediate NO(%)	Resistant NO(%)	Susceptible NO (%)	Intermediate NO(%)	Resistant NO (%)	
Ampicillin	7 (10%)	2 (2.58%)	61 (87.14%)	15 (50%)	4 (13.33%)	11(36.66%)	<0.001
Amikacin	50 (71.42%)	14 (20%)	6 (8.51%)	19 (63.33%)	6 (20%)	5 (16.66%)	0.4
Ceftazidime	32 (45.72%)	5 (7.14%)	33 (47.14%)	16 (53.33%)	(0%)	14 (46.66%)	0.5
**Gentamicin**	56 (80%)	2 (2.58%)	12 (17.14%)	15 (50%)	7 (23.33%)	8 (26.66%)	0.008
**Ciprofloxacin**	24 (34.29%)	1 (1.42%)	45 (64.29%)	21 (70%)	4 (13.33)	5 (16.66%)	<0.001
**Co-trimoxazole**	11 (15.71%)	0 (0%)	59 (84.28%)	18 (60%)	5 (16.66%)	7 (23.33%)	<0.001
**Nalidixic acid**	17 (24.28%)	3 (4.28%)	50 (71.42%)	14 (46.66%)	4 (13.33%)	12 (40%)	0.005
**Cefotaxime**	28 (40%)	0 (0%)	42 (60%)	15 (50%)	0 (0%)	15 (50%)	0.3

*p < 0.05 is considered statistically significant.

## References

[1] Kulkarni R, Dhakal BK, Slechta ES, Kurtz Z, Mulvey MA, Thanassi DG (2009). Roles of putative type II secretion and type IV pilus systems in the virulence of uropathogenic Escherichia coli. PLoS One.

[2] Ejrnæs K (2011). Bacterial characteristics of importance for recurrent urinary tract infections caused by Escherichia coli. Dan. Med. Bull..

[3] Gangoue-Pieboji J, Koulla-Shiro S, Ngassam P, Adiogo D, Ndumbe P (2006). Antimicrobial activity against gram negative bacilli from Yaounde Central Hospital, Cameroon. Afr. Health. Sci..

[4] Boucher Y, Labbate M, Koenig JE, Stokes H (2007). Integrons: mobilizable platforms that promote genetic diversity in bacteria. Trends Microbiol..

[5] Mazel D (2006). Integrons: agents of bacterial evolution. Nat. Rev. Microbiol..

[6] Rowe-Magnus DA, Mazel D (2001). Integrons: natural tools for bacterial genome evolution. Curr. Opin. Microbiol..

[7] Franklin R, Matthew A, Jeff A, Michael N, George M (2012). Performance Standards for Antimicrobial Susceptibility Testing; Twenty-Second Informational Supplement. CLSI Document M100-S22 Wayne, PA: Clinical and Laboratory Standards Institute.

[8] Roe MT, Vega E, Pillai SD (2003). Antimicrobial resistance markers of class 1 and class 2 integron-bearing Escherichia coli from irrigation water and sediments. Emerg. Infect. Dis..

[9] Sunde M (2005). Prevalence and characterization of class 1 and class 2 integrons in Escherichia coli isolated from meat and meat products of Norwegian origin. J. Antimicrob. Chemother..

[10] Kahlmeter G (2003). Prevalence and antimicrobial susceptibility of pathogens in uncomplicated cystitis in Europe. The ECO· SENS study. Int. J. Antimicrob. Agents.

[11] Farshad S, Ranjbar R, Anvarinejad M, Shahidi MA, Hosseini M (2010). Emergence of multi drug resistant strains of Escherichia coli isolated from urinary tract infection. Iran. J. Public Health.

[12] Khan AU, Zaman MS (2006). Multiple drug resistance pattern in Urinary Tract Infection patients in Aligarh. Biomed. Res..

[13] Mashouf R, Babalhavaeji H, Yousef J (2009). Urinary tract infections: bacteriology and antibiotic resistance patterns. Indian Pediatr..

[14] Molina-López J, Aparicio-Ozores G, Ribas-Aparicio RM, Gavilanes-Parra S, Chávez-Berrocal ME, Hernández-Castro R, Manjarrez-Hernández HA (2011). Drug resistance, serotypes, and phylogenetic groups among uropathogenic Escherichia coli including O25-ST131 in Mexico City. J. Infect. Dev. Ctries.

[15] Wu CT, Lee HY, Chen CL, Tuan PL, Chiu CH (2016). High prevalence and antimicrobial resistance of urinary tract infection isolates in febrile young children without localizing signs in Taiwan. J. Microbiol. Immunol. Infect..

[16] Yüksel S, Öztürk B, Kavaz A, Özçakar ZB, Acar B, Güriz H, Aysev D, Ekim M, Yalçinkaya F (2006). Antibiotic resistance of urinary tract pathogens and evaluation of empirical treatment in Turkish children with urinary tract infections. Int. J. Antimicrob. Agents.

[17] Hussain A, Ewers C, Nandanwar N, Guenther S, Jadhav S, Wieler LH, Ahmed N (2012). Multiresistant uropathogenic Escherichia coli from a region in India where urinary tract infections are endemic: genotypic and phenotypic characteristics of sequence type 131 isolates of the CTX-M-15 extended-spectrum-β-lactamase-producing lineage. Antimicrob. Agents Chemother..

[18] Sahm DF, Thornsberry C, Mayfield DC, Jones ME, Karlowsky JA (2001). Multidrug-Resistant Urinary Tract Isolates of Escherichia coli: Prevalence and Patient Demographics in the United States in 2000. Antimicrob. Agents Chemother..

[19] Yun KW, Kim DS, Kim W, Lim IS (2015). Molecular typing of uropathogenic Escherichia coli isolated from Korean children with urinary tract infection. Korean J. Pediatr..

[20] Dehbanipour R, Rastaghi S, Sedighi M, Maleki N, Faghri J (2016). High prevalence of multidrug-resistance uropathogenic Escherichia coli strains, Isfahan, Iran. J. Nat. Sci. Biol. Med..

[21] Japoni A, Gudarzi M, Farshad S, Basiri E, Ziyaeyan M, Alborzi A, Rafaatpour N (2008). Assay for integrons and pattern of antibiotic resistance in clinical Escherichia coli strains by PCR-RFLP in Southern Iran. Jpn. J. Infect. Dis..

[22] Khoramrooz SS, Sharifi A, Yazdanpanah M, Hosseini SAAM, Emaneini M, Gharibpour F, Gharibpour F, Parhizgari N, Mirzaii M, Zoladl M, Khosravani SA (2016). High Frequency of Class 1 Integrons in Escherichia coli Isolated From Patients With Urinary Tract Infections in Yasuj, Iran. Iran. Red. Crescent Med. J..

[23] Shams F, Hasani A, Rezaee MA, Nahaie MR, Hasani A, Haghi MHSB, Pormohammad A, Arbatan AE (2015). Carriage of class 1 and 2 integrons in quinolone extended-spectrum-β-lactamase-producing and multi drug resistant E coli and K pneumoniae: high burden of antibiotic resistance. Adv. Pharm. Bull..

[24] Farshad S, Japoni A, Hosseini M (2008). Low distribution of integrons among multidrug resistant E coli strains isolated from children with community-acquired urinary tract infections in Shiraz. Iran. Pol. J. Microbiol..

[25] Rezaee MA, Sheikhalizadeh V, Hasani A (2011). Detection of integrons among multi-drug resistant (MDR) Escherichia coli strains isolated from clinical specimens in northern west of Iran. Braz. J. Microbiol..

[26] Ranjbaran M, Zolfaghari M, Japoni-Nejad A, Amouzandeh-Nobaveh A, Abtahi H, Nejad M, Ghaznavi-Rad E (2013). Molecular investigation of integrons in Escherichia coli and Klebsiella pneumoniae isolated from urinary tract infections. J. Mazandaran Univ. Med. Sci..

[27] Al-Assil B, Mahfoud M, Hamzeh AR (2013). First report on class 1 integrons and trimethoprim-resistance genes from dfrA group in uropathogenic E coli (UPEC) from the aleppo area in Syria. Mob. Genet. Elements.

[28] Muhammad I, Uzma M, Yasmin B, Mehmood Q, Habib B (2011). Prevalence of antimicrobial resistance and integrons in Escherichia coli from Punjab, Pakistan. Braz. J. Microbiol..

[29] Solberg OD, Ajiboye RM, Riley LW (2006). Origin of class 1 and 2 integrons and gene cassettes in a population-based sample of uropathogenic Escherichia coli. J. Clin. Microbiol..

[30] Lin Z, Lai YM, Zaw MT (2015). Prevalence of class 1, class 2, class 3 integrons in antibiotic resistant uropathogenic Eschericheria coli isolates. Indian J. Med. Res. Pharm. Sci..

